# Supplementary material for: The interactome of intact mitochondria by cross-linking mass spectrometry provides evidence for coexisting respiratory supercomplexes
Source: Mol Cell Proteomics. 2017 Dec 8;17(2):216–32. doi: 10.1074/mcp.RA117.000470 (PMC5795388; doi:10.1074/mcp.RA117.000470)
Supplement: Supplemental Data [file supp_RA117.000470_133922_0_supp_23978_fzffwf.zip › spectra_annotation/mito_spectra_annotation/2-1-2-1-8-1.pdf]

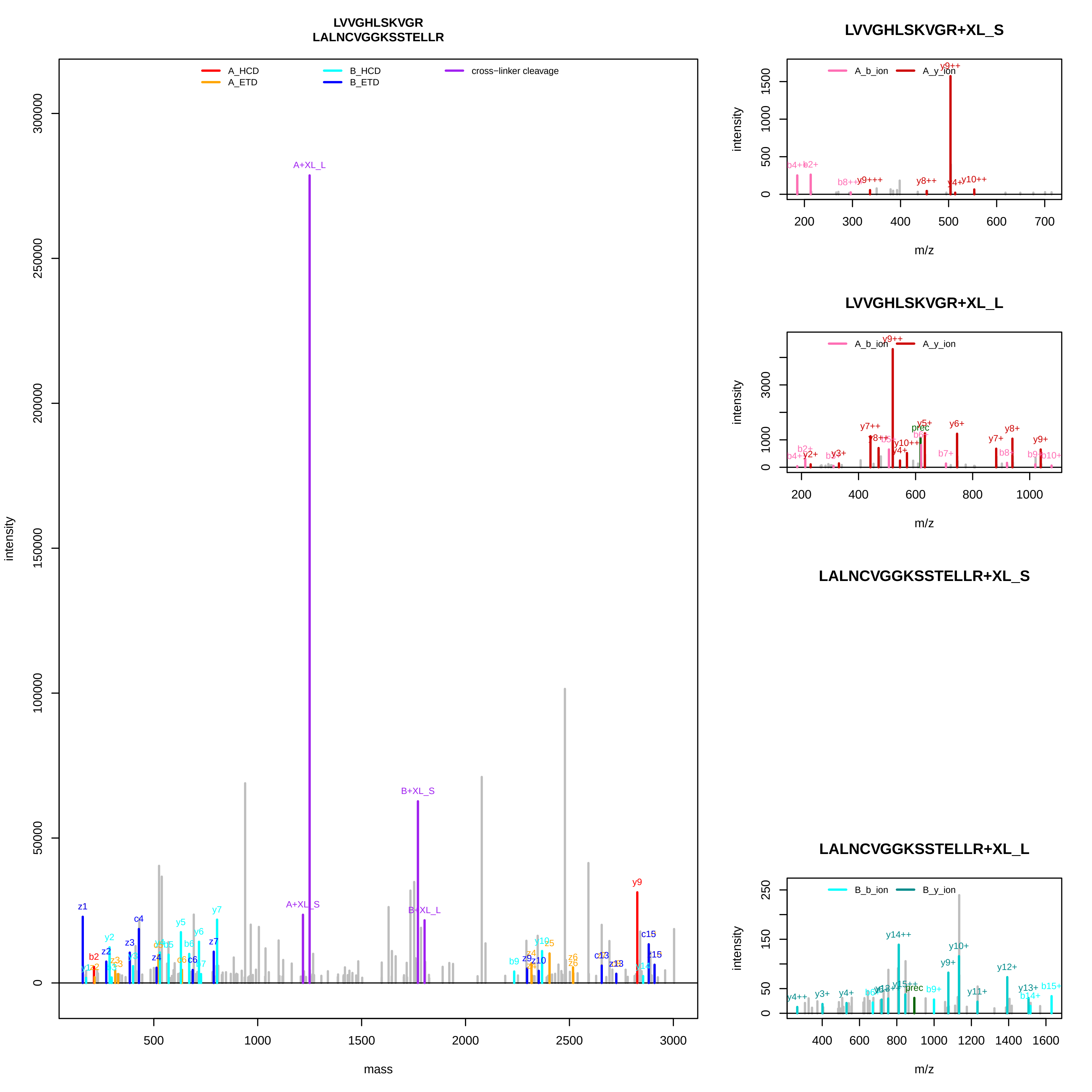
LVVGHLSKVGR
LALNCVGGKSSTELLR
A_HCD
A_ETD
B_HCD
B_ETD
cross−linker cleavage
A+XL_L
B+XL_S
A+XL_S
B+XL_L
y9
c15
z15
c13
z13
z1
c4
z3
z2
y2
b2
y7
y5
y6
z7
b9
z9
z10
y10
intensity
mass
0
50000
100000
150000
200000
250000
300000
500
1000
1500
2000
2500
3000
LVVGHLSKVGR+XL_S
A_b_ion
A_y_ion
y9++
b4++
b2+
b8++
y9+++
y8++
y4+
y10++
intensity
m/z
0
500
1000
1500
200
300
400
500
600
700
LVVGHLSKVGR+XL_L
A_b_ion
A_y_ion
y9++
y7++
y8++
prec
y5+
y6+
y10++
y7+
y8+
y9+
b7+
b8+
b9+
b10+
b2+
y2+
y3+
intensity
m/z
0
1000
3000
200
400
600
800
1000
LALNCVGGKSSTELLR+XL_S
LALNCVGGKSSTELLR+XL_L
B_b_ion
B_y_ion
y14++
y10+
y9+
y12+
y15++
prec
b9+
y11+
y13+
b14+
b15+
y4++
y3+
y4+
intensity
m/z
0
50
150
250
400
600
800
1000
1200
1400
1600
